# Extrapolating rock physicochemical properties from discrete sample data using computer vision

**DOI:** 10.1093/pnasnexus/pgag204

**Published:** 2026-06-05

**Authors:** Lewis J C Grant, Jennifer Lington, Elliot J Carter, Damon A H Teagle, Thomas M Belgrano, Rosalind M Coggon

**Affiliations:** UCD School of Earth Sciences, University College Dublin, Dublin D04 V1W8, Ireland; School of Ocean and Earth Science, National Oceanography Centre Southampton, University of Southampton, Southampton SO14 3ZH, UK; School of Ocean and Earth Science, National Oceanography Centre Southampton, University of Southampton, Southampton SO14 3ZH, UK; School of Life Sciences, Keele University, Newcastle-under-Lyme ST5 5BG, UK; School of Ocean and Earth Science, National Oceanography Centre Southampton, University of Southampton, Southampton SO14 3ZH, UK; UCD School of Earth Sciences, University College Dublin, Dublin D04 V1W8, Ireland; School of Ocean and Earth Science, National Oceanography Centre Southampton, University of Southampton, Southampton SO14 3ZH, UK

**Keywords:** computational geoscience, core logging, computer vision, drilling

## Abstract

Quantifying the lithological, physical, and chemical properties of geological samples requires experts to generate records of visual material properties, followed by targeted sampling to build a comprehensive geological understanding of the material. This understanding, therefore, is grounded in a significant amount of time on the part of the annotator to reliably extrapolate physicochemical information gained from relatively few discrete samples. It is not feasible for an expert to visually describe the entire surface of a sample; leading to only a coarse level of information where large amounts of material are being scrutinized. To overcome these limitations, we leverage the descriptive power of deep learning to generate objective high spatial resolution classifications of a ∼100 m long drill core of in situ carbonate cemented ocean crust talus breccia. Our AI-generated lithological classifications are used to semiautonomously extrapolate bulk density and carbon concentration of discrete samples to the entire surface of the core at a 0.25 cm^2^ resolution. Synthetic bulk density generated shows improvement to traditional GRA measurements, and our estimate of carbon content confirms the importance of oceanic talus breccia in global biogeochemical cycles.

Significance statement Geoscientists traditionally rely on sparse, destructive measurements to characterize heterogeneous rocks. Here, we show that computer vision can automate the classification of drill core imagery to project laboratory-derived properties across unsampled intervals, creating continuous, sub-centimeter synthetic records. This method converts visual data into objective, quantitative datasets, enhancing the efficiency of geological characterization. Using carbonate-cemented ocean crust talus breccia as a case study, we refine bulk density estimates and highlight the significance of talus breccia for carbon sequestration. Most importantly, this framework establishes a scalable paradigm for reconstructing continuous subsurface property records from existing core repositories worldwide, offering a powerful tool to audit large-scale databases and accelerate the discovery of geological and biogeochemical trends.

## Introduction

In the geosciences, it is standard for researchers to generate a detailed visual classification of a given lithological unit to inform an effective discrete sampling strategy representative of the unit as a whole. This strategy is often dictated by the volume of material available, but also the financial and time cost associated with the suite of desired physical and chemical measurements. The issue of sample abundance is exacerbated when discrete analyses require destructive techniques that further reduce the amount of material available for subsequent studies. Even in situations where sample material is abundant, it is often advantageous to analyze a representative subset to reduce time and resources requirements. Consequently, it is common for much of the material collected from the field to remain underutilized. Without direct analysis, the physical and chemical properties of this material can only be statistically inferred from its lithological similarity to the discrete sample subset analyzed in the laboratory (1). This makes detailed descriptive lithological archives essential for extrapolating the physicochemical information gained from a discrete sample dataset.

Detailed lithological logging is fundamental to both scientific and commercial drilling campaigns because they recover material whose spatial and stratigraphic context must be reconstructed indirectly. This applies whether core recovery is low, where careful cataloging and targeted sampling help maximize the limited material; or high, where sampling must be sufficiently representative of large recovered volumes. When scientific cores are recovered, proportions of rock types, alteration features, fractures, and minerals within them are recorded in detail (2). Traditionally, this task has been carried out by dedicated experts who painstakingly create descriptive and quantitative logs of the recovered material over the course of days, weeks, or even months, depending on the detail required, amount of core under analysis, and resources available (3, 4). This manual process, while thorough and rooted in the expertise of geologists, is time-consuming, labor-intensive, and subject to biases. Individual experts may interpret features differently (5–9), especially when distinctions between lithological features are subtle or visual quantifications of feature abundance, that are inherently subjective, are required. Fortunately, when digital images of the core are available, deep learning and computer vision methods offer novel approaches of automating and standardizing the objective quantification of visible characteristics (10, 11). Unlike human experts, deep learning computer vision models are not susceptible to fatigue or subjective interpretation, ensuring consistent results across datasets. Moreover, these models can generate large volumes of descriptive data in a fraction of the time required by geologists.

Previous applications of computer vision for the analysis of optical core images have focused on presenting the descriptive accuracy of a given method (11–14) and demonstrating its ability to objectively quantify features in the core (10). In this study, we go beyond simple optical quantification by using semantic computer vision classifications to robustly extrapolate physicochemical data derived from discrete samples to intervals where no samples were taken. Here we use the spatially guided semisupervised method *GeoCLR* for image classification due to its proven applicability to a variety of optical imagery (10, 15, 16). However, in response to the continued and rapid advancement of image classification methods (17), our approach of generating “synthetic” physicochemical logs is intended to be flexible to the user’s choice of image classifier and discrete sample dataset. Where image quality is sufficient to distinguish lithological textures, classification algorithms can be used to label the image based on lithology. In addition, these labels can be paired with colocated discrete measurements to create a robust calibration dataset that allow extrapolation of measured properties to the entire image. Using this approach we generate “synthetic” high-resolution records of bulk density, primary porosity and carbon content for a section of ancient carbonate-cemented talus breccia from the Mid-Atlantic Ridge. At the modern Mid-Atlantic ridge axis, faulting causes talus piles to accumulate at the foot of the axial ridge valley walls (18, 19), and the discovery of >100 m of talus breccia during International Ocean Discovery Program (IODP) Expedition 390 suggests that such deposits are a common feature of the seafloor that remain preserved on the vast ridge flanks. Faulting, and therefore breccia accumulation, can occur at all spreading rates (20), though thick carbonate-cemented talus breccia deposits have not yet been discovered on fast spreading crust.

Ocean crust covers ∼66 of the globe, and the total area occupied by slow spread crust in the modern oceans is $1.09\times 10^{8}\;{\mathrm{km}}^{2}$ (21). Modern ocean crust is created at a rate of $3.4\;{\mathrm{km}}^{2}\;{\mathrm{year}}^{-1}$ (22), during which magma degassing releases $0.47\text{–}2.09\times 10^{12}$ moles of ${\mathrm{CO}}_{2}$ to the oceans and atmosphere (23). Therefore, every new km^2^ of crust produced releases $14\text{–}61\times 10^{10}$ moles of ${\mathrm{CO}}_{2}$. Using a parametric bootstrapping approach (Monte Carlo simulation), Ref. (20) suggest an average talus breccia thickness of $18_{-15}^{+14}$ m if it were to be spread evenly over all modern slow-spread crust. They also calculate an average breccia ${\mathrm{CO}}_{2}$ content of 7.5 wt%. Building on this work, we demonstrate how deep learning, combined with a suite of discrete samples, can be used to improve the accuracy and reproducibility of these global carbon assessments. By transforming optical imagery and geochemical data into continuous downhole records, we present how to more precisely quantify the heterogeneity of carbonate storage and refine our understanding of the ocean crust’s role as a long-term carbon sink.

## Geological setting and dataset

**Figure 1:**
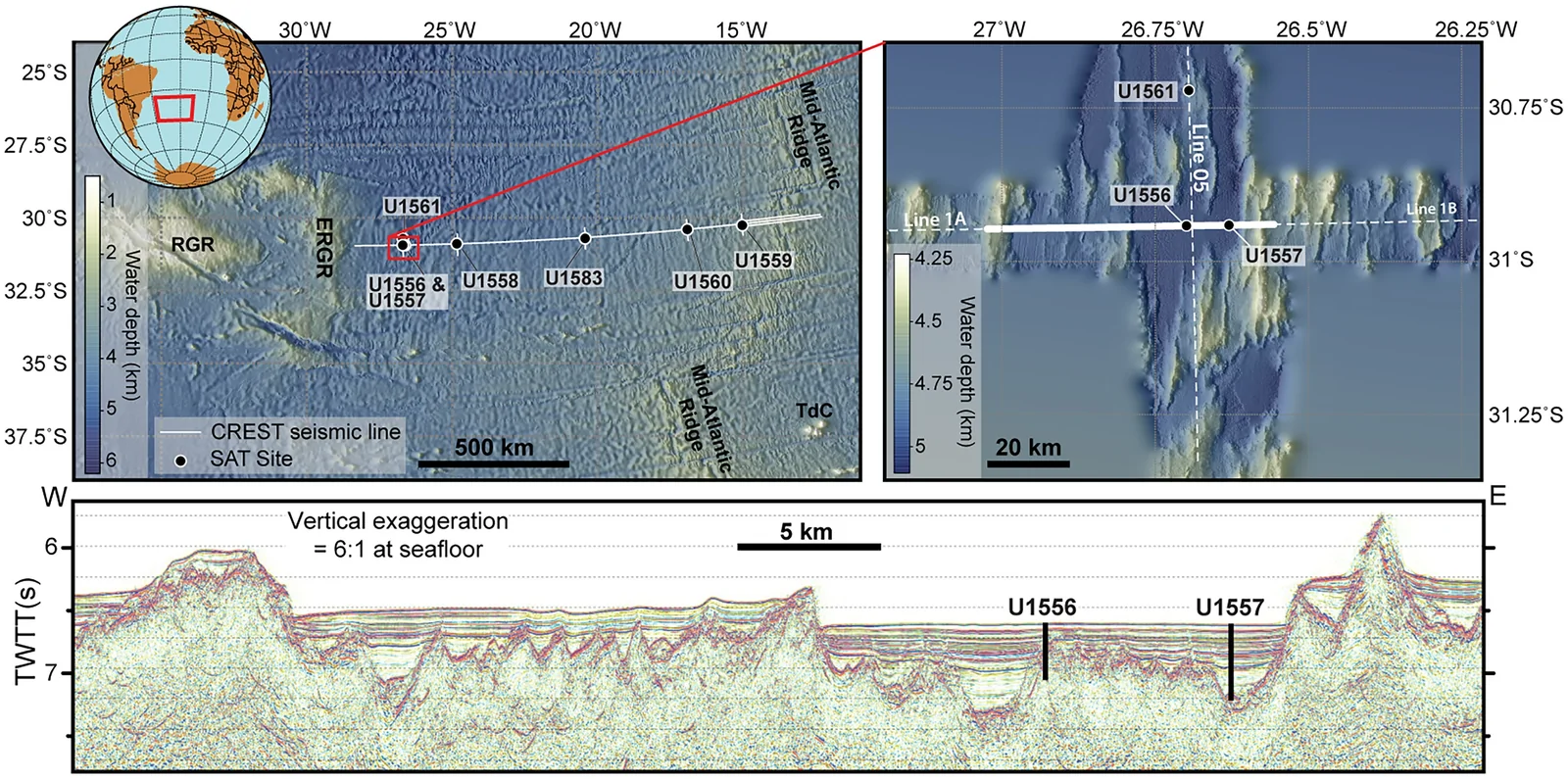
Top: left—bathymetry (24) of SAT area in the South Atlantic Ocean, showing the locations of the SAT drill site (black circles); right—the 61 Ma SAT study area, where Sites U1557 and U1556 are located in the same sediment-filled basin (3). White lines=CREST seismic reflection profiles (25), RGR=Rio Grande Rise, ERGR=eastern Rio Grande Rise, TdC=Tristan de Cunha. Bottom: CREST seismic reflection profile, parallel to the SAT, showing the locations of SAT Site U1557 adjacent to a Mid-Atlantic Ridge-parallel fault and site U1556 on the adjacent faulted basement high; portion of CREST line shown indicated by solid white line in study area map (top right). TWTT,two−way travel time. Modified after Ref. (20).

**Figure 2:**
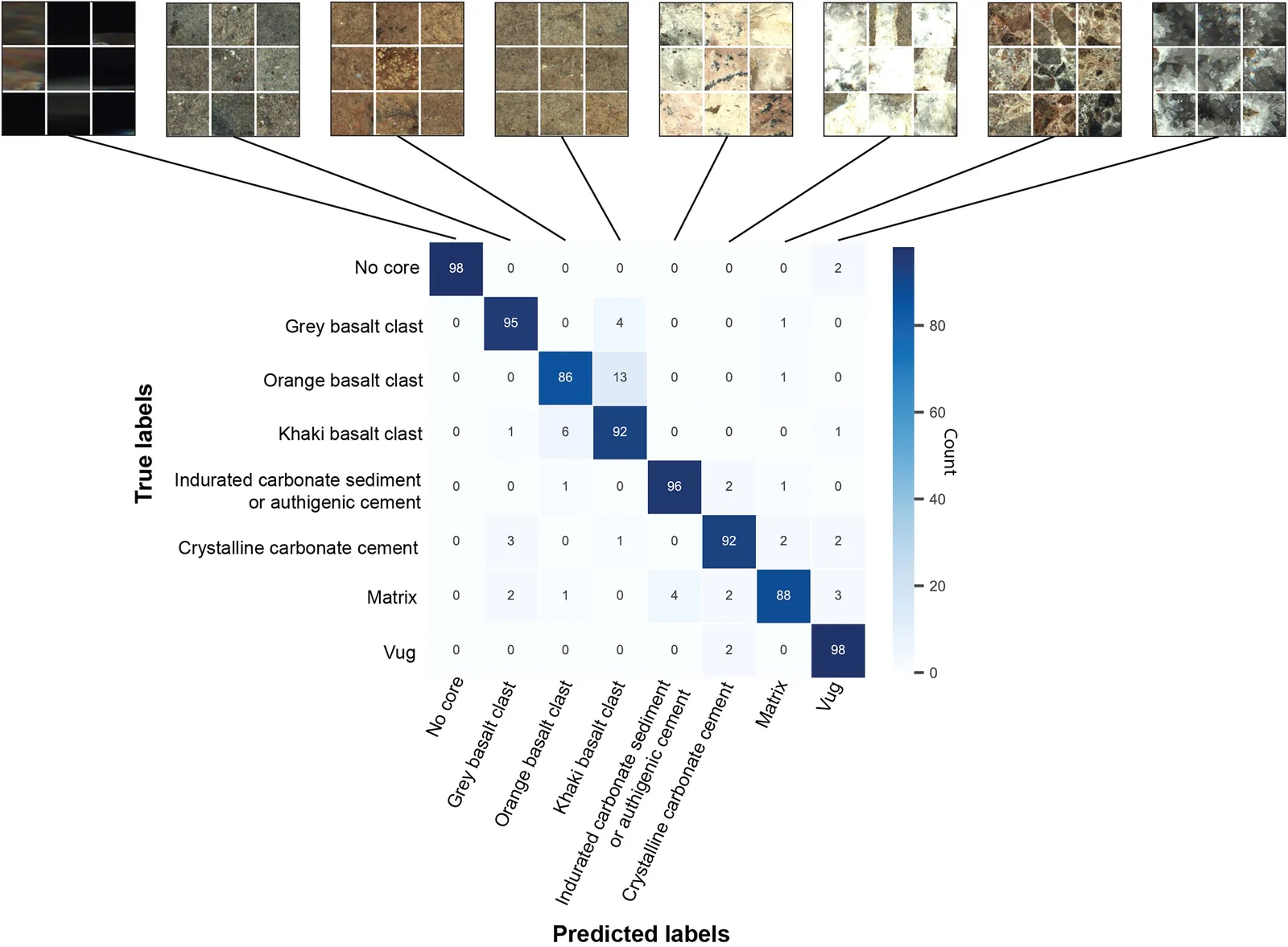
Confusion matrix summarizing the performance of the GeoCLR classification model against the 800 (100 per class) expert labeled validation image set. The matrix displays the number of correct and incorrect predictions made by the model. Rows represent the actual classes, while columns represent the predicted classes. The diagonal cells indicate correct predictions, while off-diagonal cells indicate misclassifications. The heat map visualizes the number of classifications that occurred for each actual-predicted class pair, with count indicating the frequency of occurrences. Shaded cells running diagonally indicate good agreement between expert and model-generated labels. Nice example image patches for each class are shown above the matrix.

**Figure 3:**
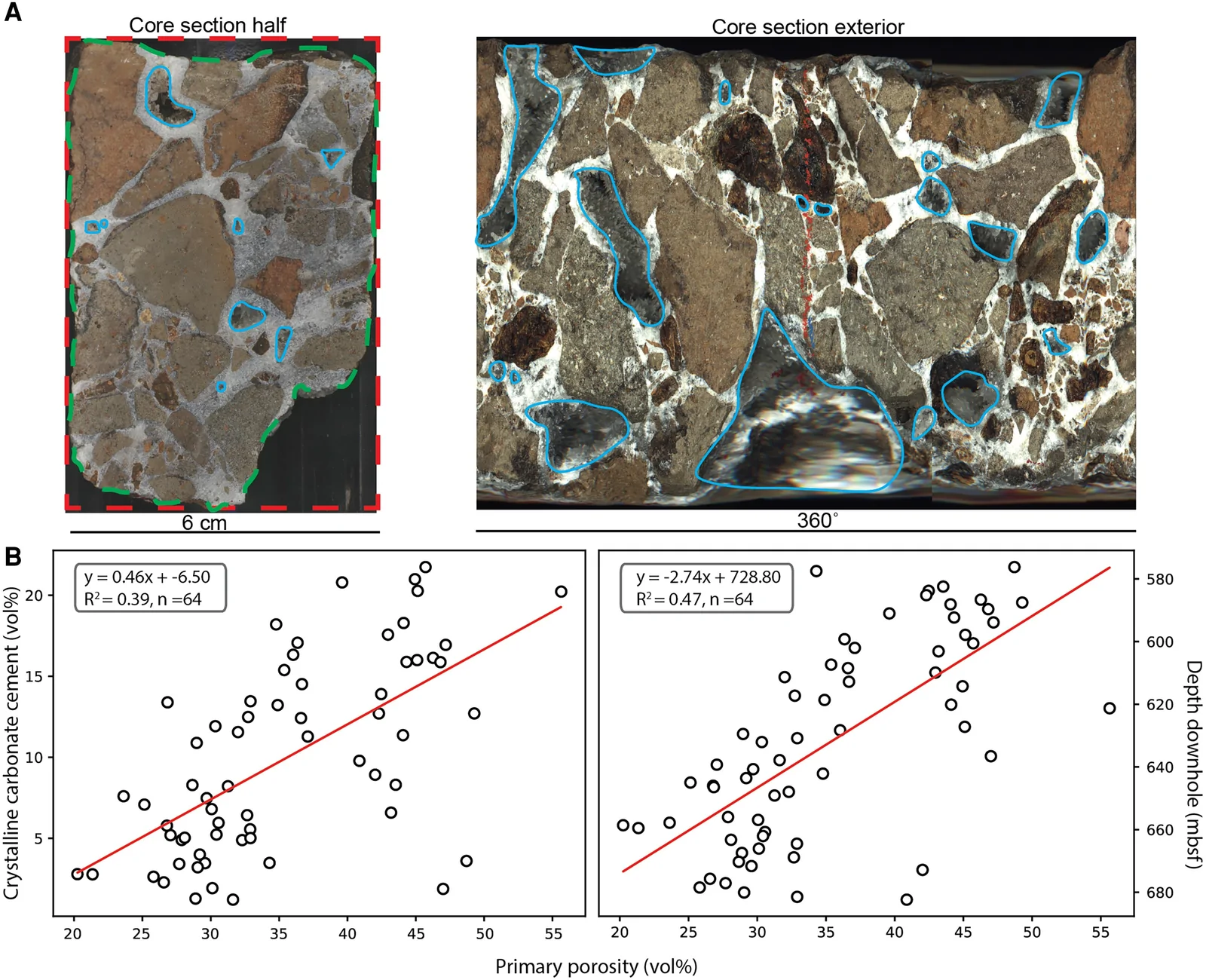
A) Macroscopic porosity (vugs) are outlined in blue on images of both the cut surface of the archive half (left) and core exterior (right). The archive half was used to determine component abundance during IODP Expedition 390; however, the area that was quantified (bounded by green dashed line) did not include uneven core edges—many of which were actually primary porosity that GeoCLR was capable of identifying in this study using the core exterior images. This figure illustrates the difficulty of directly comparing classifications made from different surfaces of the same material. B) The primary porosity of each core section versus its crystalline carbonate cement abundance (left) and depth downhole (right), with linear regression lines shown in red.

All cores analyzed here were recovered from IODP Site U1557, on the South Atlantic Transect (SAT). The SAT comprises an age transect of five primary drill sites along a single crustal flow line at $∼31^{\circ }$ S on the western flank of the southern Mid-Atlantic Ridge (MAR) drilled during IODP Expeditions 390C, 395E, 390, and 393 (3). Igneous basement at Site U1557 formed 60.7 Ma at a slow spreading rate (∼13.5 mm/year half-rate) (3). Site U1557 is located ∼1250 km west of the MAR at a water depth of ∼5 km within a 2 km wide (perpendicular to spreading direction) sub-basin to the eastern edge of a larger 17.5 km fault-bounded sedimentary basin adjacent to an uplifted, faulted basement block, where another basement hole at Site U1556 was drilled (Fig. 1) (26). Normal faults that bound these basins formed subparallel to the MOR and drilling at Site U1557 sought to investigate the impact of variable sediment thickness in fault-bounded basins on the alteration of the underlying crust (3, 27, 28). At Site U1556, 340 m of volcanic upper basement was cored including pillow lavas, hyaloclastite breccias, and massive flows, similar to slow-spreading oceanic crust at other sites (3, 29). In contrast, basement drilling at Site U1557 penetrated 10 m of basaltic talus breccia in Hole U1557B, followed by a further 110 m of similar material in Hole U1557D; the underlying lava flows of the oceanic crust proper were not reached, and the full thickness of the breccia is not known. Overlying sediments are 564 m thick, with those at the sediment–basement interface containing middle Paleocene planktic foraminifera and calcareous nannoplankton. This indicates that breccia deposition occurred shortly after crustal accretion, likely during near-axial uplift along valley wall high angle normal faults (26). Consequently, this thick breccia deposit records the erosive, and subsequent depositional history of a midocean ridge fault system and contains a complex mixture of altered basalt clasts, indurated calcareous sediment, crystalline calcite cement and authigenic zeolite+calcite cement (Fig. 2). The sediment, cements, and fine volcanic material partially fill the primary interclast porosity, with substantial primary porosity still open as macroscopic pores (vugs; Fig. 3) (20).

In this study, we focus on the longer Hole U1557D, which shipboard core descriptions categorize as a single depositional unit divided into two subunits; subunit 1b (567.9–574.22 m below seafloor (mbsf)) where the matrix is dominantly composed of recrystallized calcareous pelagic sediment and subunit 1c (584.52–682.66 mbsf) where the matrix is dominantly composed of authigenic zeolite+calcite cement (3). Within a “transition zone” between ∼628 and 638 mbsf deposits become more compacted, and hence porosity and crystalline carbonate cement abundance decreases, whereas authigenic carbonate+zeolite cement abundance increases in the lower portion of subunit 1c.

Immediately following recovery, cores had their external surface digitally imaged at a resolution of 40 pixels/mm using a DMT CoreScan3 with a line scanning camera that imaged the core as it was rotated (30, 31). These cores are strongly cemented with high recovery rates (65.3%) and consequently of the 71.2 m recovered 70.8 m were consolidated enough to be imaged on the DMT Corescan3 (3, 30). Following standard scientific ocean drilling procedures, each ≤9 m long core recovered was divided into ≤1.5 m long core sections comprising multiple core “pieces” (3). Pieces were defined as individual or multiple lengths of core that showed continuity, and where a break in continuity occurs within a section a divider was added to indicate the start/end of a new piece (30). All images used here are of individual pieces and after imaging, the cores were split lengthwise into an archive and working half. Formal descriptions generally focus on the cut surface of the archive half and the outer core surfaces receive only qualitative inspection (3). The complex arrangement of the breccia components did not allow for high resolution descriptions, and the proportions of given features or rock types was visually estimated at the decimeter-to-meter scale. Though these methods are currently standard during a drilling campaign, they do not account for inconsistency and biases between annotators. In the case of Site U1557, this limitation was avoided as all igneous and alteration petrology core descriptions were carried out by the same annotators. However, this also limited the resolution of descriptions due to time constraints.

## Methods

### Objective quantification of breccia components

Each core exterior image was segmented into 0.5×0.5 cm ($0.25\;{\mathrm{cm}}^{2}$) patches yielding $∼4.3\times 10^{5}$ image patches. This patch size was selected to be small enough to reduce overlap of class boundaries within a given patch, but also to be large enough that the diagnostic features of classes are discernible. All patches were classified using a spatially guided, semisupervised computer vision framework developed by Ref. (10) specifically for semantic classification of 3D drill core imagery. Their workflow adapts the *GeoCLR* method of Ref. (16) by learning image representations (latent embeddings) through selfsupervised contrastive training (see Supplementary material for details).

Following encoder training, latent embeddings (*h*) for all images together provide an unlabeled feature space for the entire dataset. In order to generate labels for all image patches, an expert labeled subset of 3,100 images was used to train a support vector machine (SVM) with a radial basis function kernel. The trained SVM was then applied to the entire dataset, assigning pseudolabels to unlabeled images by extrapolating the learned decision boundaries in the feature space. While the SVM itself is supervised, this pseudolabeling step yields a semisupervised pipeline by leveraging predictions on the unlabeled portion of the data. All classes match the same lithological groupings used when discrete samples were selected for shipboard moisture and density (MAD) and postcruise carbon analysis (20, 26). Classes (n) included; altered basalt clasts (gray, orange, and khaki), authigenic zeolite+carbonate cement, matrix, crystalline carbonate cement, indurated carbonate sediment and open porosity (vugs). All basalt within Hole U1557D has undergone alteration to some extent, and the color of the clast represents a continuum of alteration facies (gray → khaki → orange) defined by the extent of oxidation (26). Images classified as matrix contain millimeter scale basalt clasts and/or volcanic glass fragments and crystalline carbonate and/or authigenic zeolite+carbonate cement, with clasts occupying on average 68 area% (see Supplementary methods).

Visually it is not possible to distinguish authigenic zeolite+carbonate cement and indurated carbonate sediment as both appear as reddish yellow to pale brown fine grained material. However, shipboard thin section descriptions revealed that above 587.7 mbsf this fine grained component is microfossil-bearing indurated carbonate sediment, whereas below it is authigenic zeolite+carbonate cement (26). Therefore, whether an image was classified as authigenic zeolite+carbonate cement or indurated carbonate sediment depended on its depth within the borehole, as both are visually indistinguishable on the core surface. Expert generated labels (*M*) were not class-balanced, but at least ∼300 images per class (M/n) were labeled for SVM training. This minimum number of labels was selected following previous applications of *GeoCLR* to classify geological imagery that showed a flattening of performance between M/n=100 and M/n=300 (10).

In order to quantify the accuracy of the classification method, an additional independent validation subset of 100 labeled images from each of the eight classes were gathered to compare model predictions to expert labels. This validation subset was kept separate from the 3,100 image training set used for classification, though all images were still used during the self-supervised feature extraction of *GeoCLR*. Therefore, the performance of the *GeoCLR* method presented here should be considered dataset specific and is not intended to insinuate its potential performance during future applications. A macro average $f_{1}$ score across the eight classes was chosen as our preferred performance metric because it provides a balanced measure of the model’s accuracy by considering both precision (the proportion of correctly identified positive instances) and recall (the proportion of actual positives correctly identified) (32). This makes $f_{1}$ scores especially useful for evaluating imbalanced datasets, where an overemphasis on either false positives or false negatives given by the model classifier can skew results.

### Bulk density

During IODP Expeditions it is standard practice to measure the physical properties of recovered cores, and the bulk density of Site U1557 core was measured at a 10 cm resolution down-core using a gamma ray attenuation (GRA) logger, as well as via MAD analysis of $36\times 8\;{\mathrm{cm}}^{3}$ discrete samples. Here, we use the objective output of *GeoCLR* to extrapolate a 0.5 cm resolution (ie effectively continuous) record of bulk density downhole from data obtained from discrete shipboard samples. This is possible because *GeoCLR* enabled the entire core surface to be classified into the lithological classes assigned to discrete samples, enabling properties to be estimated for the entire core, assuming true physical and chemical properties are represented within the distribution of values obtained during laboratory analyses of similar material (1). Here, we chose to reconstruct the bulk density as it can be easily validated against the GRA density obtained during Expedition 390, as these measurements were made on the same material at a similar spatial scale. Although downhole measurements made using wireline tools also yield continuous records, their direct comparison with properties of recovered cores is challenging because (i) they analyze borehole wall material which may have different properties to cores from the same depth, given the cm-scale heterogeneities in the formations; and (ii) core recovery is typically incomplete, hence depth correlations between cores and wireline data can be challenging.

As the image patches comprise a 2D image of a 3D ($360^{\circ }$) surface of core, unlike the 2D images of the split core surface, they capture the 3D geometry of the breccia. Therefore, the volume proportion of each breccia component is assumed here to be equivalent to the area proportion of each class as observed at a given depth in the images. The bulk density for each 0.5 cm depth interval ($\rho_{d}$) is given by:


(1) $$\rho_{d}=\sum_{i}\rho_{i}v_{i-d}=\sum_{i}\rho_{i}\frac{N_{i-d}}{N_{d}},$$

where $\rho_{i}$ is the bulk density of breccia component *i* (Table 1), $v_{i-d}$ is the volume proportion of each component in that depth interval (*d*), and $N_{i-d}$ is the number of image patches of component *i* in the interval. $N_{d}$ is the total number of patches in interval *d* (normalized to remove nongeological classes). Bulk density of pure crystalline carbonate cement is $2.71\;g/{\mathrm{cm}}^{2}$. For matrix, indurated carbonate sediment, authigenic zeolite+carbonate cement, and altered basalt clasts the mean of shipboard MAD-derived bulk density analyses for samples of each class, respectively, were used (Table 1).

**Table 1.** Mean bulk density and ${\mathrm{CO}}_{2}$ concentration values used to generate the synthetic logs in this study, derived from MAD and combustion analysis of discrete samples, respectively.

Class	Bulk density $g/{cm}^{3}$	${CO}_{2}$ wt%	Shipboard vol%	GeoCLR vol%	Precision	Recall	$f_{1}$ score
Gray altered basalt clast	2.73±0.08	1±1.2			0.94	0.95	0.95
Khaki altered basalt clast	2.52±0.11	0.8±0.7	81.8±5.2	66±5.5	0.84	0.92	0.88
Orange altered basalt clast	2.52±0.11	0.8±0.7			0.91	0.86	0.89
Indurated carbonate sediment	2.40±0.06	41.9±2.4	2.2±5.4	1.3±3.9	0.96	0.96	0.96
Authigenic zeolite + carbonate cement	2.32±0.07	26±5.8	6±5.1	14±4.7			
Matrix	na	na	na	na	0.95	0.88	0.91
Crystalline carbonate	2.71	44.01	9.1±2.7	10.8±5.5	0.94	0.92	0.93
Vugs	0	0	0.9±0.7	7.8±4.7	0.92	0.95	0.95
No core	na	na	na	na	1.00	0.98	0.99
Macro average:					0.93	0.93	**0.93**

Mean shipboard estimates for breccia components generated visually by experts during IODP Expedition 390 are also shown alongside the same estimates made semiautonomously using *GeoCLR* in this study. All vol% values here are core averages weighted by core interval lengths to account for variable core recovery. Errors here represent 1SD. Precision, recall, and $f_{1}$ scores for each class identified using *GeoCLR* summarize its performance for each class and macroaverages show its overall perforce across all classes.

### Carbon concentration

Bulk carbon concentrations of Hole U1557D authigenic zeolite+carbonate cement and altered basalt clasts were measured by Ref. (20), who combined them with shipboard core descriptions to estimate the carbon content of the breccias. Here, we build upon the dataset of Ref. (20), by clustering their carbon concentration data based on the basalt alteration facies (gray, khaki, or orange) targeted during shipboard MAD analysis. Additional carbon measurements of indurated carbonate sediment and authigenic zeolite+carbonate cement samples were also measured to ensure that both geochemical and physical properties information were available for all representative lithologies. All carbon concentrations were measured using a LECO carbon and sulphur (CS744) analyser at the National Oceanography Centre Southampton following the analytical procedures of Ref. (20) (see Supplementary material).

Thin sections and hand sample descriptions, shipboard X-ray diffraction, and chemical analyses all confirm that the crystalline carbonate throughout Hole U1557D is pure, or near pure, ${\mathrm{CaCO}}_{3}$ (20). Furthermore, as the overlying sediments were calcareous, the recrystallized pelagic carbonate sediment within the upper ∼20 m of the breccia is near pure ${\mathrm{CaCO}}_{3}$ with a bulk density of $2.4\;g/{\mathrm{cm}}^{3}$ (20). In contrast, below 20 m downhole this interstitial microcrystalline material contained a mix of authigenic zeolite(phillipsite)+carbonate cement and ${\mathrm{CaCO}}_{3}$, with petrographic descriptions confirming it comprised 50 vol% ${\mathrm{CaCO}}_{3}$, consistent with the 63 wt% ${\mathrm{CaCO}}_{3}$ measured using elemental analysis (20, 26).

The ${\mathrm{CO}}_{2}$ content of each 0.5 cm depth interval $([{\mathrm{CO}}_{2}{]}_{d}$) was calculated following:


(2) $$[{\mathrm{CO}}_{2}{]}_{d}=\sum_{i}[{\mathrm{CO}}_{2}{]}_{i}\cdot m_{i-d},$$

where [${\mathrm{CO}}_{2}$]*_i_* is the ${\mathrm{CO}}_{2}$ content (wt%) of breccia component *i* (Table 1), and $m_{i-d}$ is the mass proportion of component *i* in *d*, given by:


(3) $$m_{i-d}=\frac{\rho_{i}}{\rho_{d}}\cdot v_{i-d}.$$

The ${\mathrm{CO}}_{2}$ content of each core $\left([{\mathrm{CO}}_{2}{]}_{c}\right)$ is then given by:


(4) $$[{\mathrm{CO}}_{2}{]}_{c}=\frac{\sum_{d}[{\mathrm{CO}}_{2}{]}_{d}\cdot m_{d}}{\sum_{d}m_{d}},$$

where $m_{d}$ is the total mass of the core at depth interval *d* ($=\rho_{d}v_{d}$), and $v_{d}$ is the total volume of core in depth interval *d*. Furthermore, the mean ${\mathrm{CO}}_{2}$ content of the cored breccia deposit from Hole U1557D, weighted by drilled core interval lengths ($D_{c}$) to normalize for variable core recovery is given by;


(5) $$[{\mathrm{CO}}_{2}{]}_{\mathrm{breccia}}=\frac{\sum_{c}[{\mathrm{CO}}_{2}{]}_{c}\cdot \rho_{c}\cdot D_{c}}{\sum_{c}\rho_{c}\cdot D_{c}}.$$

## Results and discussion

### GeoCLR performance and class abundance

Basalt (including the estimated volume of clasts in matrix patches) represent 57–73 vol% of the recovered cores with a mean abundance of 66±5.5 vol% (±1SD), weighted by core interval length to normalize for variable core recovery. This is lower than the shipboard estimate of 82.6 vol%, which in part we attribute to human overestimation biases that commonly arise when visual estimates of abundance are made (6–8). The tendency for this overestimation is greatest for the most abundant features in a scene (9), and it is therefore not surprising that it manifests here for the most abundant breccia component. The accuracy of visual cover estimates decreases with the increasing scale at which estimations are made (33), though improvements can be made if the scene is subdivided (34). As all class abundances in this study are made by combining values from many sub-cm patches, they are likely more accurate than those made at a decimeter scale. However, the images used here captured whole-round core exteriors, which represent volumetrically more core, and a different surface than split core section surfaces described during Expedition 390 (Fig. 3), so 1:1 agreement is not expected.

As identified in the shipboard logs, there is no overall systematic change in the proportion of altered basalt clast types downhole. However, where fresher gray clasts occur, they tend to be in discrete intervals. Khaki alteration is more prevalent than orange alteration throughout the core, and both show opposing trends in abundance compared to fresher gray clasts. Shipboard core descriptions indicate that khaki and orange alteration account for 20–70 vol% and 4–40 vol% of each core, respectively. In contrast, *GeoCLR*-derived estimates are much lower at 1–24 vol% and 0–13 vol%, respectively.

Indurated carbonate sediment and authigenic carbonate+zeolite cement have a combined mean abundance of 15.3±6.1 vol% (±1SD) per core, 7.7 vol% higher than shipboard estimates, whereas *GeoCLR*-derived estimates of pure crystalline carbonate cement abundance are similar to visual estimates at 10.8±5.5 vol% (±1SD) (Table 1). Based on *GeoCLR*-derived estimates, the cores contain on average 7.8±4.7 vol% macroscopic open porosity; with an overall decrease in open porosity of 0.2 vol%/m downhole. Our open porosity estimate is an order of magnitude larger than the shipboard estimate of 0.9±0.7 vol%, in part due to *GeoCLR* identifying vugs where dog-toothed textures indicate the solid core surface comprises minerals precipitated on the margins of open porosity. Such dog-toothed carbonate is common at ends of core pieces, suggesting that breaks in core represent unrecovered void space. Additionally, the 3D core exterior images used here capture open void spaces that were not fully surrounded by rock on the split surface of core sections, hence were not logged during Expedition 390 (Fig. 3). The resultant higher *GeoCLR*-based estimates of open porosity abundance may account for some of the difference between our results and shipboard-derived clast abundance estimates.

### Primary porosity and borehole electrical resistivity

Hole U1557D breccias are dominantly clast supported and the interstitial space between clasts, now filled with cement, indurated sediment, and matrix was primary porosity. Therefore, the original “primary” porosity of the breccia pile immediately following its deposition can be inferred from the volume of the core not occupied by basalt clasts. Based on the lithological classifications of *GeoCLR*, the breccia originally contained 21–39 vol% primary porosity, with a mean of 30 vol% weighted by core interval length. Downhole the volume of original “primary” porosity correlates with the volume of crystalline carbonate cement that has precipitated within pores since deposition (Fig. 3). This correlation highlights the pervasiveness of interconnected primary porosity within a talus breccia pile, and supports the view that they have a large surface area for fluid-rock reaction and accommodation space for subsequent precipitation of hydrothermal minerals (20). Although, accommodation space decreases with depth where the breccia has been compacted beneath the weight of overlying material (Fig. 3).

At Site U1557, the base of the breccia pile was not reached, so it is possible that it is thicker than the recovered ∼110 m. However, linear regression of primary porosity with depth indicates that there would be minimal remaining porosity deeper than ∼730 m downhole, hence more vigorous fluid flow is likely restricted to the upper ∼160 m of a breccia pile blanketed by sediment. Electrical resistivity of the borehole wall was measured following coring of Hole U1557D as a proxy for the connectivity of the subsurface pore network and the degree of cementation (26). Broadly, borehole resistivity decreases downhole, which if taken in isolation would suggest an increase in pore connectivity with depth. Despite the resistivity trend, the downhole reduction in core-observed porosity implies that the formation actually becomes more occluded, not better connected, with depth.

**Figure 4:**
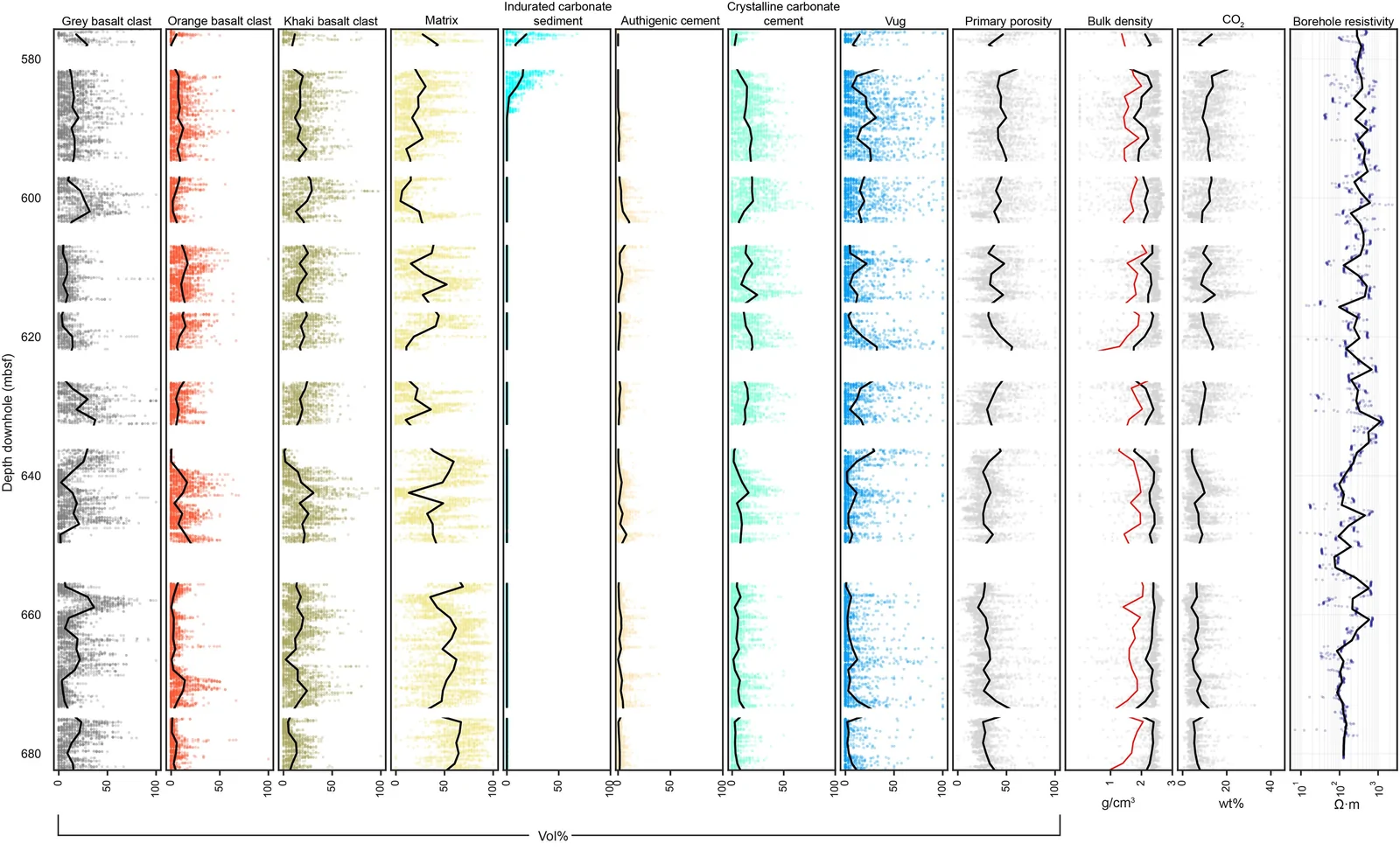
From left to right, the first eight panels show the *GeoCLR*-derived downhole plots of volume percent for breccia components, whereas the next three gray panels show the calculated primary porosity, bulk density (red line=1.5 m binned average for GRA data) and ${\mathrm{CO}}_{2}$ concentration generated using *GeoCLR*-derived classification and discrete sample data. The far right panel shows true (RT) borehole wall electrical resistivity data collected using a High-Resolution Laterolog Array (HRLA) during IODP Expedition 390. Dots show the data at a 0.5 cm downhole resolution and black lines represent a 1.5 m binned average. As per IODP protocols, recovered core material is curated from the top of each cored interval, with incomplete recovery resulting in breaks in the section downhole. Breaks in the data represent these nonrecovered intervals, as well as intervals where core was too fragmented to digitally image using the DMT Corescan3.

Direct comparison between wireline resistivity and core-derived porosity is hindered by the breccia’s heterogeneity at the cm-scale; however, by binning our high-resolution lithological data into 1.5 m depth averages, we can reconcile core observations with the broader trends captured by the downhole logs (Fig. 4). The reduction in macroscopic porosity downhole suggests that it is not the primary driver of formation resistivity. Instead, the abundance of phillipsite-rich matrix shows the most statistically significant relationship to borehole resistivity ($r^{2}=0.2$, Pvalue=0.001). A clear increase in matrix abundance below 630 mbsf coincides with a sustained decrease in resistivity (Fig. 4), suggesting that the development of a conductive, zeolite-rich matrix network dominates the electrical response of the formation. Consequently, borehole resistivity in this setting tracks the intensity of matrix alteration rather than pore-network connectivity, necessitating caution when using electrical logs to infer the hydrologic properties of cemented oceanic crust.

### High-resolution computationally derived bulk density

During Expedition 390, bulk density measurements for all cores were conducted using a gamma ray attenuation (GRA) sensor (3). In general, GRA is most effective when applied to soft sediments, where the assumption of a uniform core diameter is generally valid, as sediment cores typically fill their core liner completely, minimizing measurement artifacts and yielding reliable bulk density estimates. In contrast, use of GRA on hard rock cores presents significant challenges as cores commonly exhibit irregularities such as fractures, voids, uneven surfaces, or incomplete core recovery, resulting in partial filling of the core liner that leads to a density underestimation via GRA.

Figure 4 shows 1.5 m binned averages through the GRA and *GeoCLR*-derived downhole bulk density, the latter created using only images and a limited number of representative discrete samples (n=36). Based on our GeoCLR-derived results, the average bulk density ($\rho_{\mathrm{bulk}}$) of the Hole U1557D breccia is $2.24\;g/{\mathrm{cm}}^{3}$. The two datasets display a strong positive correlation (Pearson’s r=0.73, $P\text{-value}=5.04\times 10^{-9}$), however, the GeoCLR-derived bulk density is consistently higher than the GRA data and has a mean offset of $0.49\pm 0.17\;g/{\mathrm{cm}}^{3}$ (±1SD). This offset appears to be less pronounced where bulk density is higher, and the ratio between GRA and *GeoCLR*-derived bulk densities can be used to estimate the amount to which GRA is underestimating for a given bulk density measurement.

### High-resolution computationally derived carbon concentration

Degassing of basaltic magma during the production of ocean crust is an integral part of the carbon cycle, currently releasing $1.32_{-0.85}^{+0.77}\times 10^{12}$ mol ${\mathrm{CO}}_{2}\;{\mathrm{year}}^{-1}$ to the modern oceans and atmosphere (23). Subsequent precipitation of secondary ${\mathrm{CaCO}}_{3}$ minerals from seawater-derived hydrothermal fluids counteracts this outgoing flux by trapping carbon back into the lithosphere. The low temperature hydrothermal fluid flux through the vast ridge flanks is many orders of magnitude greater than through high-temperature axial hydrothermal systems (35, 36), and much of this sequestration occurs off-axis at low temperatures (37, 38). Hydrothermal circulation is driven by heat from the cooling crust (38), with fluids flowing through permeable pathways that may remain active throughout the crust’s entire life cycle, in part due to persistent reactivation of fractures promoted by plate cooling (39). As a consequence of this continuous reactivation of fluid pathways, veins increase in width, but not abundance (39), and over time the crust accumulates more ${\mathrm{CaCO}}_{3}$ as a function of its age by filling fractures to form veins, replacing primary minerals and mesostasis, cementing breccias and filling other open spaces, such as vesicles (37, 38). Previous estimates place the modern crustal carbon sink at $∼2.4\times 10^{12}\;\mathrm{mol}$  ${\mathrm{CO}}_{2}\;{\mathrm{year}}^{-1}$ (37), indicating the ocean crust is a net sink for ${\mathrm{CO}}_{2}$. These estimates include ${\mathrm{CaCO}}_{3}$ that cements hyaloclastite/volcanic breccias within lava stratigraphy of the volcanic upper crust, however, they do not include thick breccia piles like that recovered at Site U1557, which accumulate on top of the volcanic stratigraphy.

Table 1 shows the average ${\mathrm{CO}}_{2}$ concentrations obtained by combustion analysis in this study with all results given in this section referring to the mean concentrations ±1SD. Using Eq. 2, these mean values, in combination with a ${\mathrm{CO}}_{2}$ concentration of 44 wt% for crystalline calcite cement, allowed us to calculate ${\mathrm{CO}}_{2}$ concentration for each 0.5 cm depth increment down hole ($[{\mathrm{CO}}_{2}{]}_{d}$) (Figure 4). Downhole shifts in ${\mathrm{CO}}_{2}$ concentration broadly follow the abundance of crystalline carbonate cement due to its high carbon content, and there is a clear downward decrease in ${\mathrm{CO}}_{2}$ concentration at the lithological subunit 1b–1c transition. This lithological transition is marked by a gradual decrease in remaining porosity, indicating that crystalline carbonate cement abundance, and hence ${\mathrm{CO}}_{2}$ concentration, is dependent on the accommodation space (primary porosity) for hydrothermal precipitates. Overall, recovered cores from Hole U1557D contain 4.8–12.6 wt% ${\mathrm{CO}}_{2}$ with a mean concentration of 9.1±2.4 wt% ${\mathrm{CO}}_{2}$ weighted by core interval length and core bulk density. This estimate is ∼2 wt% higher than that estimated by Ref. (20) using coarser expert-generated logging data, hence ocean crust talus breccia appears to be a more significant carbon sink than previously calculated using traditional techniques.

As the U1557D breccia has a bulk density of $2.24\;g/{\mathrm{cm}}^{3}$, this global breccia pile on slow-spread crust would have a mass of $4.4_{-3.7}^{+3.4}\times 10^{18}$ kg (1σ-equivalent). If the Site U1557 breccias are representative of this reservoir, it would contain ∼9 wt% ${\mathrm{CO}}_{2}$, equating to a total ${\mathrm{CO}}_{2}$ concentration of $3.8_{-3.2}^{+3.0}\times 10^{17}$ kg, or $3.5_{-2.9}^{+2.7}\times 10^{9}$ kg (1σ-equivalent) of breccia-hosted ${\mathrm{CO}}_{2}\;\mathrm{per}\;{\mathrm{km}}^{2}$ of ocean crust. Therefore, this carbon sink alone has the potential capacity to offset 2 to 102% of the ${\mathrm{CO}}_{2}$ released by volcanic degassing during crustal production. Furthermore, the high volume of primary porosity remaining in the breccia, even after 60 Ma of traversing the seafloor, indicates there may be as much as 8 vol% open porosity for continued ${\mathrm{CaCO}}_{3}$ precipitation. Assuming seawater can continue to penetrate the crust throughout its life span, particularly in regions with high basement relief and discontinuous sediment blanketing, our results suggest that an average $1\;m^{3}$ of talus breccia of similar age to Site U1557 could store an additional $0.08\;m^{3}$ (∼95 kg) of ${\mathrm{CO}}_{2}$, assuming all void space was filled with ${\mathrm{CaCO}}_{3}$.

## Conclusions

This study demonstrates how objective quantification of the volumetric abundance of geological features via computer vision provides a robust framework for assessing lithological, physical, and chemical variations within rocks. Physicochemical data from discrete samples were gathered and averaged in groups representative of major rock types within a ∼100 m carbonate cemented talus breccia deposited by mass wasting at a slow spreading mid-ocean ridge. Lithological descriptions, bulk density, and carbon concentration of these breccias have previously been recorded using traditional manual logging, line scanning for physical properties, and discrete sample analyses. Here, we demonstrate that this critical information can accurately be determined by extrapolating physicochemical properties of discrete samples to material where no samples were taken, using computer vision. Firstly, training of the semisupervised deep learning framework *GeoCLR* generated semantic classifications for each $0.25\;{\mathrm{cm}}^{2}$ of the core surface, with classes selected to match the lithological criteria used to select discrete samples. These classifications then allowed us to calculate the abundance of each lithology, and their combined physicochemical properties at each 0.5 cm depth interval downhole, providing the first dataset where these lithological, physical and chemical data can be compared at a consistent spatial scale. Our continuous *GeoCLR*-derived estimate of bulk density correlates well with bulk density derived from GRA analyses but overcomes the GRA measurement-related issues that typically result in underestimation of bulk density in hard rock cores. Similarly, our reassessment of the carbon concentration of these talus breccias show they are a significant sink for ${\mathrm{CO}}_{2}$ and confirm their importance in long term biogeochemical cycling.

Image classification algorithms including those trained via supervised, unsupervised or semisupervised learning all seek to produce a dataset of labels assigned to input images. As deep learning and computer vision are rapidly evolving fields of research, there are an abundance of models and approaches to choose from, each with potentially differing performance on a dataset-by-dataset basis. In response, the methods outlined in this study can be applied and tested using any labeled image dataset where labels correspond to another property of the material/object in the image, regardless of the classification algorithm used. For images with accompanying spatial data, methods such as GeoCLR can exploit this metadata to improve their performance (10, 16). Fortunately, where spatial information is not available, many of these methods still show reliable performance provided a suitably large training dataset is available (10, 40).

In both scientific and industrial subsurface research, there is a critical need for tools that can transform legacy archives into high-resolution, quantitative datasets. The methodologies presented here enable researchers to revisit existing repositories and produce a new generation of high-fidelity interpretations, unlocking the untapped potential of historical core records to address modern geoscience challenges.

## Supplementary Material

pgag204_Supplementary_Data

## Data Availability

The MAD, carbon concentration and image classifier data underlying this article are available at https://doi.org/10.5281/zenodo.20524560. All core images used are available at https://doi.org/10.14379/iodp.proc.390393.209.2024.

## References

[1] Childress LB, Acton GD, Percuoco VP, Hastedt M. 2024. The LILY database: linking lithology to IODP physical, chemical, and magnetic properties data. Geochem Geophys Geosyst. 25:e2023GC011287.

[2] Alt JC, et al Hydrothermal alteration of a section of upper oceanic crust in the Eastern Equatorial Pacif: a synthesis of results from site 504 (DSDP LEGS 69, 70, and 83, and ODP LEGS 111, 137,140, and 148). In: *Proc Ocean Drilling Program, Scientific Results*. Vol. 148. IODP, College Station, TX, USA, 1996. p. 417–434.

[3] Coggon RM, *et al*., South Atlantic Transect IODP Expedition 390/393 Scientists. South Atlantic transect. In: *Proc Int Ocean Discov Program*. Vol. 390/393. IODP, College Station, Texas, USA, 2024.

[4] Kelemen PB, Matter JM, H Teagle DA, Coggon JA, Oman Drilling Project Scientists. Oman drilling project: scientific drilling in the Samail ophiolite, sultanate of Oman. In: *Proc Oman Drilling Project*. IODP, College Station, TX, US, 2020.

[5] Curtis EJ, et al 2024. Improving coral monitoring by reducing variability and bias in cover estimates from seabed images. Prog Oceanogr. 222:103214.

[6] Finn PG, Udy NS, Baltais SJ, Price K, Coles L. 2010. Assessing the quality of seagrass data collected by community volunteers in Moreton Bay Marine Park, Australia. Environ Conserv. 37:83–89.

[7] Greene MR . 2016. Estimations of object frequency are frequently overestimated. Cognition. 149:6–10. 10.1016/j.cognition.2015.12.011 26774103

[8] Olmstead MA, Wample R, Greene S, Tarara J. 2004. Nondestructive measurement of vegetative cover using digital image analysis. HortScience. 39:55–59.

[9] Osorio K, Puerto A, Pedraza C, Jamaica D, Rodríguez L. 2020. A deep learning approach for weed detection in lettuce crops using multispectral images. AgriEngineering. 2:471–488.

[10] Grant LJC, et al 2024. Leveraging spatial metadata in machine learning for improved objective quantification of geological drill core. Earth Space Sci. 11:e2023EA003220.

[11] Mamode HI, Hampson GJ, John CM. 2025. Do more with less: exploring semi-supervised learning for geological image classification. Appl Comput Geosci. 25:100216.

[12] Alzubaidi F, Mostaghimi P, Swietojanski P, Clark SR, Armstrong RT. 2021. Automated lithology classification from drill core images using convolutional neural networks. J Pet Sci Eng. 197:107933.

[13] Fu D, Su C, Wang W, Yuan R. 2022. Deep learning based lithology classification of drill core images. PLoS One. 17:e0270826. 10.1371/journal.pone.0270826 PMC9249224 35776744

[14] Zhang PY, Sun JM, Jiang YJ, Gao JS. Deep learning method for lithology identification from borehole images. In: *79th EAGE Conference and Exhibition 2017*. European Association of Geoscientists & Engineers, Bunnik, The Netherlands, 2017. p. 1–5.

[15] Li T, et al Predicting multi-level socioeconomic indicators from structural urban imagery. In: *Proceedings of the 31st ACM International Conference on Information & Knowledge Management*. Association for Computing Machinery, New York, NY, USA, 2022. p. 3282–3291.

[16] Yamada T, Prügel-Bennett A, Williams SB, Pizarro O, Thornton B 2022. GeoCLR: georeference contrastive learning for efficient seafloor image interpretation. Field Robot. 2:1134–1155.

[17] Kalokhe S, et al Computer vision advancement with vision transformers: a comprehensive review. In: *2024 5th International Conference on Intelligent Communication Technologies and Virtual Mobile Networks (ICICV)*. IEEE, 2024. p. 447–453.

[18] Allerton S, Murton BJ, Searle RC, Jones M. 1995. Extensional faulting and segmentation of the Mid-Atlantic Ridge north of the Kane Fracture Zone (24 00’ N to 24 40’ N). Mar Geophys Res. 17:37–61.

[19] Cannat M, Mangeney A, Ondréas Hélène, Fouquet Y, Normand A. 2013. High-resolution bathymetry reveals contrasting landslide activity shaping the walls of the Mid-Atlantic Ridge axial valley. Geochem Geophys Geosyst. 14:996–1011.

[20] Coggon RM, et al, Transect IODP Expedition 390/393 Scientists. 2025. A geological carbon cycle sink hosted by ocean crust talus breccias. Nat Geosci. 18:1279. 10.1038/s41561-025-01839-5 PMC12685744 41378368

[21] Seton M, et al 2020. A global data set of present-day oceanic crustal age and seafloor spreading parameters. Geochem Geophys Geosyst. 21:e2020GC009214.

[22] Rowley DB . 2002. Rate of plate creation and destruction: 180 Ma to present. Geol Soc Am Bull. 114:927–933.

[23] Le Voyer M, et al 2019. Carbon fluxes and primary magma ${\mathrm{CO}}_{2}$ contents along the global mid-ocean ridge system. Geochem Geophys Geosyst. 20:1387–1424.

[24] Ryan WBF, et al 2009. Global multi-resolution topography synthesis. Geochem Geophys Geosyst. 10:Q03014.

[25] Reece R, Estep J. 2019. Processed MCS (PSTM) data from the Mid-Atlantic Ridge (MAR) to the Rio Grande Rise, South Atlantic Ocean, acquired by the R/V Marcus G. Langseth in 2016 (MGL1601).

[26] Coggon RM, et al., South Atlantic Transect IODP Expedition 390/393 Scientists. Site U1557. In: Proc Int Ocean Discov Program. Vol. 390/393. IODP, College Station, TX, USA, 2024.

[27] Christeson GL, et al 2020. South Atlantic transect: variations in oceanic crustal structure at 31 S. Geochem Geophys Geosyst. 21:e2020GC009017.

[28] Estep J, Reece B, Christeson GL, Kardell DA, Carlson RL. 2021. 70 million years of seafloor spreading and magmatism in the South Atlantic. Earth Planet Sci Lett. 574:117173.

[29] Flower MFJ, Ohnmacht W, Robinson PT, Marriner G, Schmincke HU. Lithologic and chemical stratigraphy at Deep Sea Drilling Project sites 417 And 418. In: *Proc of Ocean Drilling Program: Initial Report*. Vol. 939. 1980.

[30] Grant LJC, et al Data report: high-resolution digital imaging of whole-round hard rocks collected during IODP Expeditions 390C, 395E, 390, and 393, South Atlantic Transect, using a DMT CoreScan3. In: Proc Int Ocean Discov Program. Vol. 39. 2024.

[31] LECO: 744 series, combustion analysis for carbon and sulfur . https://www.leco.com/products/744-series/ [Accessed 06/01/2025], 2025.

[32] Handelman GS, et al 2019. Peering into the black box of artificial intelligence: evaluation metrics of machine learning methods. Am J Roentgenol. 212:38–43. 10.2214/AJR.18.20224 30332290

[33] Moody A, Woodcock C. 1994. Scale-dependent errors in the estimation of land-cover proportions: implications for global land-cover datasets. Photogramm Eng Remote Sensing. 60:585–594.

[34] Dethier MN, Graham ES, Cohen S, Tear LM. 1993. Visual versus random-point percent cover estimations: “objective” is not always better. Mar Ecol Prog Ser. 96:93–100.

[35] Mottl MJ, Geoffrey Wheat C. 1994. Hydrothermal circulation through mid-ocean ridge flanks: fluxes of heat and magnesium. Geochim Cosmochim Acta. 58:2225–2237.

[36] Nielsen SG, et al 2006. Hydrothermal fluid fluxes calculated from the isotopic mass balance of thallium in the ocean crust. Earth Planet Sci Lett. 251:120–133.

[37] Alt JC, H Teagle DA. 1999. The uptake of carbon during alteration of ocean crust. Geochim Cosmochim Acta. 63:1527–1535.

[38] Gillis KM, Coogan LA. 2011. Secular variation in carbon uptake into the ocean crust. Earth Planet Sci Lett. 302:385–392.

[39] Evans AD, et al 2025. Ocean crustal veins record dynamic interplay between plate-cooling-induced cracking and ocean chemistry. Earth Planet Sci Lett. 650: 119116.

[40] Liang C, et al 2025. Investigating location-regularised self-supervised feature learning for seafloor visual imagery [preprint], arXiv, arXiv:2509.06660, https://arxiv.org/abs/2509.06660.

