# Correction to: Extrapolating rock physicochemical properties from discrete sample data using computer vision

**DOI:** 10.1093/pnasnexus/pgag325

**Published:** 2026-09-29

This is a correction to: Lewis J C Grant, Jennifer Lington, Elliot J Carter, Damon A H Teagle, Thomas M Belgrano, Rosalind M Coggon, Extrapolating rock physicochemical properties from discrete sample data using computer vision, *PNAS Nexus*, Volume 5, Issue 6, June 2026, pgag204, https://doi.org/10.1093/pnasnexus/pgag204

In the originally published version of this article, co-author Damon A. H. Teagle was erroneously listed as affiliated to Keele University instead of University of Southampton.

This error has been corrected.

